# Traditional eye medicine use by newly presenting ophthalmic patients to a teaching hospital in south-eastern Nigeria: socio-demographic and clinical correlates

**DOI:** 10.1186/1472-6882-9-40

**Published:** 2009-10-24

**Authors:** Boniface Ikenna Eze, Chimdi Memnofu Chuka-Okosa, Judith Nkechi Uche

**Affiliations:** 1 Department of Ophthalmology, University of Nigerian Teaching Hospital (UNTH), PMB 01129, Ituku-Ozalla, Enugu, Nigeria

## Abstract

**Background:**

This study set out to determine the incidence, socio-demographic, and clinical correlates of Traditional Eye Medicine (TEM) use in a population of newly presenting ophthalmic outpatients attending a tertiary eye care centre in south-eastern Nigeria.

**Methods:**

In a comparative cross-sectional survey at the eye clinic of the University of Nigeria Teaching Hospital (UNTH), Enugu, between August 2004 - July 2006, all newly presenting ophthalmic outpatients were recruited. Participants' socio-demographic and clinical data and profile of TEM use were obtained from history and examination of each participant and entered into a pretested questionnaire and proforma. Participants were subsequently categorized into TEM- users and non-users; intra-group analysis yielded proportions, frequencies, and percentages while chi-square test was used for inter-group comparisons at P = 0.01, df = 1.

**Results:**

Of the 2,542 (males, 48.1%; females, 51.9%) participants, 149 (5.9%) (males, 45%; females, 55%) used TEM for their current eye disease. The TEMs used were chemical substances (57.7%), plant products (37.7%), and animal products (4.7%). They were more often prescribed by non-traditional (66.4%) than traditional (36.9%) medicine practitioners. TEMs were used on account of vision loss (58.5%), ocular itching (25.4%) and eye discharge (3.8%). Reported efficacy from previous users (67.1%) and belief in potency (28.2%) were the main reasons for using TEM. Civil servants (20.1%), farmers (17.7%), and traders (14.1%) were the leading users of TEM. TEM use was significantly associated with younger age (p < 0.01), being married (p < 0.01), rural residence (p < 0.01), ocular anterior segment disease (p < 0.01), delayed presentation (p < 0.01), low presenting visual acuity (p < 0.01), and co-morbid chronic medical disease (p < 0.01), but not with gender (p = 0.157), and educational status (p = 0.115).

**Conclusion:**

The incidence of TEM use among new ophthalmic outpatients at UNTH is low. The reasons for TEM use are amenable to positive change through enhanced delivery of promotive, preventive, and curative public eye care services. This has implications for eye care planners and implementers. To reverse the trend, we suggest strengthening of eye care programmes, even distribution of eye care resources, active collaboration with orthodox eye care providers and traditional medical practitioners, and intensification of research efforts into the pharmacology of TEMs.

## Background

Worldwide, the last two decades have witnessed a phenomenal increase in the prevalence of use of TEM [1,2]. This is despite that, till date, there is no sound scientific evidence to justify the use of TEMs in treating eye diseases [3]. The resort to patronage of TEM has been variously attributed to ignorance, barriers to access primary eye care services, preference, failure of conventional treatment, desire to take control over medical treatment, communication gap between patients and orthodox eye care providers, and influence of friends and relations [2,4-7].

Traditional eye medicines (TEMs) are a form of biologically-based therapies or practices that are instilled or applied to the eye or administered orally to achieve a desired ocular therapeutic effect. [3,4]. TEMs are crude or partially processed organic (plant and animal products) or in-organic (chemical substances) agents or remedies that are procured from either a traditional medicine practitioner-TMP (Synonyms: Traditional alternative Medicine Practitioner-TAMP; Traditional Healer-TH, Spiritual Healer) or non-traditional medicine practitioners which could be the patient, relation, or friend [1,2].

TEM use, either as sole first line treatment, or as adjunct used concurrently with conventional therapy has been associated with poor visual, ocular, and occasionally survival outcome of otherwise treatable eye diseases in clinical ophthalmic practice [2,8-10].

TEM- related poor ophthalmic treatment outcomes have been attributed to delay in uptake of eye care services while on first line TEM therapy; damage to ocular and or adnexal structures from intrinsic TEM toxicity or result of interaction with prescribed medications, and microbial contamination of TEM agent or procedure [2,10-12].

The lack of standardization of dosage, low purity level, and non-physiologic physico-chemical properties of TEM, and its unintended role as culture medium for pathogens account for the observed adverse effects [2,10].

Various epidemiological surveys on TEM have documented varying prevalence of TEM use, and established inconsistent associations of TEM use with diverse factors like age, gender, educational status, rural location of residence, occupation, socio-economic status, ownership of health insurance, access to eye care, and time to presentation for uptake of eye care services [1,5,13-15].

TEM use reflects the diverse eye health care needs of the population not met by the existing eye care delivery system. Consequently, to effectively rein in the tendency to use TEM, there is the need to understand the health behaviour, health literacy status, socio-economic variables, and dimensions of barrier to access eye care in the population [1,5,15].

In Nigeria's public health sector, promotive, preventive, and curative orthodox health care delivery is carried out within the frame work of Primary Health Care. The healthcare system consists of primary, secondary, and tertiary levels of care which are run by the local, state, and federal governments respectively. However, there exist an ample number of privately owned healthcare institutions that are also involved in the delivery of health services to the public. In addition, traditional(un-orthodox) medical practice, although not an officially recognised healthcare alternative, is flourishing and enjoys patronage from a broad spectrum of the society. Among Nigerians, cultural and religious beliefs and practices, especially as they relate to health, are strong and influence their health seeking behaviour often in favour of traditional medical care. The widespread use of traditional medical remedies and practices for the treatment of common diseases and ailments has, of recent, prompted the Federal Government, of Nigeria to initiate and fund research activities to identify useful traditional herbal medicines [16]. This, hopefully, would improve the quality of traditional medical services. This study was intended to determine the incidence, socio-demographic, and clinical correlates of TEM use in a clinic population of new ophthalmic outpatients at a tertiary eye care facility in South-Eastern Nigeria.

## Methods

The University of Nigeria Teaching Hospital (UNTH), Enugu, was established in 1971 and is one of the first generation public tertiary hospitals in Nigeria. The mandate of the institution includes training, provision of clinical services, and research. At UNTH, specialist clinical care is provided on outpatient or inpatient basis; however, there exists a separate GOPD where patients may initially present with or without referral letters. Patients presenting to the GOPD with minor ailments are usually treated as such and discharged while those requiring specialist care are referred, as appropriate, to specialist outpatient clinics. Patients referred with official referral letters, from sources both within and outside UNTH, present directly to the specialist outpatient clinics. At the ophthalmic outpatient clinic, promotive, preventive, curative, and rehabilitative eye care services are provided by consultant ophthalmologists, resident ophthalmologists, optometrists, and nurses.

### Questionnaire and proforma development

The instruments used for data collection consisted of a structured, open-ended, researcher administered questionnaire and a proforma (Additional file 1). To validate and ascertain the psychometric reliability of the questionnaire, it was pre-tested on twenty new ophthalmic patients with or without history of TEM use for the current eye disease. The proforma, used for recording clinical examination findings, was similarly pretested. Subsequently, observed structural defects in the questionnaire and proforma, as highlighted by the pre-tests, were corrected before used for the study.

### Study population and design

This was a comparative cross-sectional study involving all newly presenting ophthalmic patients who presented consecutively.

### Setting

Ophthalmic outpatient clinic of UNTH, Enugu; 1 August 2004 - 31 July 2006. The study questionnaire was used to collect relevant information on patient's socio-demographic characteristics, general health status, use of prescribed medicines, and TEM use pattern while the proforma was used to record their clinical details after evaluation. Based on reported use of TEM for the current eye disease episode, the study participants were categorised into two groups: TEM users, and TEM non-users.

For the TEM users, we obtained further information on the nature, prescriber, reason for use, duration of use, and route of administration of TEM. Also, we inquired whether TEM use was on-going at the time of consultation; for those who had abandoned TEM use, we sought to know the reason/s for cessation of TEM therapy.

Subsequently, each participant had a comprehensive ophthalmic examination including refraction, followed by relevant clinical and laboratory investigations, where needed, to arrive at the definitive clinical diagnosis.

During the pretest, we observed that most of the old patients, especially those with longstanding disease conditions, were unable to recall information relating to previous TEM use for their current eye disease. Consequently, to eliminate recall bias, all the old patients were excluded from the final survey.

### Ethical approval

The study was carried out in full compliance with the 1964 Helsinki declaration on research involving human subjects. The Ethical Committee of UNTH, Enugu, approved the study. Oral informed consent was obtained from the participants prior to enrollment into the study.

### Data analysis

The data obtained were analysed with the Statistical Package for Social Sciences (SPSS) software, version 12.0.

Intra-group analyses were performed to yield proportions, frequencies and percentages.

Inter-group comparisons were also performed to determine if there were significant differences in the gender, mean age, marital status, educational status, location of residence, time to presentation, co-morbid chronic medical disease, location of ocular pathology, and the best corrected visual acuity at presentation.

Statistical testing for significance of inter-group differences was performed with Epinfo, version 3.4 at p < 0.01, df = 1.

## Results

Two thousand five hundred and forty two (2,542) new eye patients consisting of 1,222 (48.0%) males and 1,238 (52.0%) females, aged between 3 months- 72 years (mean = 56.1 years ± 1.9 SD), were seen at the ophthalmic outpatient clinic of UNTH, Enugu. Of these, 149 patients comprising 67(45.0%) males and 82(55.0%) females aged between 3 months - 68 years (mean = 55.6 years ± 2.3 SD) reported TEM use (TEM users) for treating their current eye disease.

The incidence of TEM use in the study population was 5.9% (149/2,542).

The remaining 2,393 new patients comprising 1,155(48.3%) males and 1,238(51.7%) females, aged between 6 months-72 years (mean = 57.2 years ± 2.2SD) did not use TEM for treating their current ocular disease and they constituted the TEM non-user Group.

The age and sex distribution of the TEM users is shown in Table 1.

**Table 1:** Age and sex distribution of TEM users

	**Sex**		
**Age (yrs)**	**M**	**F**	**Total (%)**
0 -- 10	4	3	7(4.7)
11-20	13	12	25(16.8)
21-30	10	12	22(14.8)
31-40	7	10	17(12.4)
41-50	6	12	18(12.1)
51-60	7	13	20(13.4)
61-70	20	20	40(26.8)

Total (%)	67(45%)	82(55%)	149(100%)

The nature of TEM used by the patients is presented in Table 2. Animal products (7, 4.7%), plant products(56,37.7%) and chemical substances (86, 57.7%) were the groups of TEM used by the patients.

**Table 2:** Nature of TEM used

**TEM**	**n(%)**
**Animal products**	
Human urine	4(2.7)
Human breast milk	2(1.30
Cod liver oil	1(0.7)
**Plant products**	
Herbal extract	42(28.2)
Aloe vera product	10(6.7)
Palm wine	2(1.3)
Palm oil	1(0.7)
Bitter kola extract	1(0.7)
**Chemical substances**	
Holy water	56(37.6)
Anointed oil/Olive oil	13(8.7)
Black stone	4(2.7)
Natural (spring) water	3(2.0)
Powder	2(1.30)
Salt solution	2(1.3)
Sugar solution	2(1.3)
Forever living product	1(0.7)
Antimony	1(0.7)
Kerosene	1(0.7)
*Harcogen rub	1(0.7)

Total	149(100.0)

*Mentholated rub

The profile of TEM use is summarized in Table 3.

**Table 3:** Nature of TEM used

**Characteristic**	**n (%)**
**TEM prescriber**	
**Traditional medicine practitioner**	
Traditional Healer	33(22.1)
Clergyman (spiritual healer)	22(14.8)
**Non-traditional medicine practitioner**	
Patient	42(28.2)
Friend	30(20.1)
Relation	17(11.4)
Not specified	5(3.4)
**Duration of use (weeks)**	
≤ 1	58(39.0)
2-8	40(26.8)
9-24	24(16.1)
25-52	6(4.0)
> 52	21(14.1)
**Reason for use**	
Others benefited	100(67.1)
Belief in potency	42(28.2)
Orthodox medicine un-affordable	3(2.0)
Unaware of orthodox alternatives	2(1.3)
Unsatisfactory orthodox treatment	2(1.3)
**Route of administration**	
Instillation	126(84.6)
Oral	17(11.4)
Face wash	4(2.7)
Inhalation	2(1.3)

### TEM users

The TEM users were either married (42, 51.7%) or single (40, 48.3%).

Their occupations included civil service (30, 20.1%), farming (26, 17.7%), and trading (21, 14.1%).

Majority (115, 77.0%) of these patients had at least a formal primary education and reside in an urban area-92(62.0%). Diminution of vision (87, 58.5%), ocular itching (38, 25.4%), and eye discharge (3,3.8%) were the leading presenting complaints in the sub-group.

TEM users tended to present later than one month (131, 87.9%) after onset of their ocular complaint, which were mainly due to ocular anterior segment disease (108, 72.5%).

At presentation, majority (n = 120, 80.5%) of TEM users had stopped the use of TEM. Of these, unsatisfactory response to TEM therapy (71, 59.1%), worsening of eye condition (40, 33.3%), and advice from others (6, 5.0%) were the main reasons for abandonment of TEM use. Of the TEM users, 34(23.0%) were blind (best corrected distant visual acuity less than 3/60 in the better eye) at presentation.

### TEM non-users

The TEM non-users were either married (1,393, 58.2%) or single (1,000, 42.8%). Their occupations included civil service (473, 19.8%), farming (325, 13.6%) and trading (306, 12.8%). majority-1,603(67.0%) of the participants had a minimum of primary education while 2,105(88.0%) reside in an urban area.

Diminution of vision (1,220, 51.0%), ocular itching (675, 28.2%), and eye discharge (200, 8.4%) were the leading presenting complaints in this group.

TEM non-users tended to present within one month (1,316, 55.0%) of onset of their ocular complaints which were mainly due to anterior segment disease (1,484,62.0%).

The chronic diseases seen in the participants were systemic hypertension [(217, 8.5%): TEM users (24.8%), TEM non-users(7.5%), diabetes mellitus [(76, 3.0%): TEM users(13.4%), TEM non-users (2.4%)], and HIV/AIDS [(18,0.7%): TEM user(2.0%), TEM non-users(0.7%)] At presentation, 860(33.8%) patients comprising of TEM users (78, 52.3%) and TEM non-users (782,32.7%)were on prescribed medicines. The prescribed medicines were administered by direct application into the eye [TEM users (50, 33.6%), TEM non-users (332, 13.8%)] and orally [TEM users (50, 33.6%), TEM non-users (332, 13.8%)].

Inter- Group comparisons showed significant inter- group differences in mean age (55.6 ± 2.3 SD vs 57.2 ± 2.2 SD, p < 0.01), marital status (52.0% vs 32.0%, p < 0.01), residence (urban: 62% vs 91%, p < 0.01), time to presentation ≤1 month (88.0% vs 45.0%, p < 0.01), co-morbid chronic medical disease (24.8% vs 7.5%, p < 0.01), ocular anterior segment location of pathology (73.0% vs 22.0%, p < 0.01), and those with presenting visual acuity less than 3/60 (15% vs 3%, p < 0.01).

However, there was no significant inter-group difference in the proportion of female patients (55.0% vs 45.0%, p = 0.157), and educational status (77.0% vs 67.0%, p = 0.115).

## Discussion

Previous studies that have evaluated TEM use for eye diseases have reported various prevalence rates[1,11-15] but we have evaluated incidence of TEM use since our focus was on newly presenting ophthalmic patients. Since there was no previous report on the incidence of TEM use for eye diseases, we were constrained to compare incidence from our study with previously reported prevalence.

The 5.9% incidence of TEM use observed in this study is small when compared with 47.7% reported in India [1], 17.9% in Democratic Republic of Congo (DRC) [11], 33.8% in Malawi [13], 62.3% in Oman, and 49.0% in Tanzania [15]. The rate was however higher than 1.72% previously reported in Nigeria [2]. The variations in the rates may be attributed to the differences in the study settings, populations, and the specific use of TEM for the eye diseases. While the present study involved all newly presenting ophthalmic outpatients to a tertiary eye care centre, irrespective of the eye diseases, previous studies [1,13,14] investigated TEM use specifically in ophthalmic patients who presented with corneal ulcer. Others based their studies on a general population of ophthalmic patients [11] or specifically evaluated TEM use for ocular trauma [15]. A study previously done in Nigeria [2] was in a setting similar to ours but involved both old and new patients; thus while we reported incidence of TEM use, the study reported prevalence of TEM use. Furthermore, corneal ulceration and ocular trauma have been established to be associated with the likelihood of TEM therapy [11,13]. This might further explain the comparatively higher prevalence figures reported from studies on TEM use in corneal ulcer and ocular trauma patients.

The socio-demographic characteristics of the study participants revealed that those who presented with ocular complaints were predominantly in the productive age group. The majority of TEM users were aged forty years or older; however, their mean age was significantly less than that of non-users; there were more females than males in the TEM user Group, however, there was no statistically significant inter-group difference in the proportion of females. The age and gender distribution observed in this survey is similar to the observations in USA [5] but differed from the findings in India [1], Malawi [13] and the DRC [11]. Resort to TEM use by females and the aged has been attributed to gender and age related barriers to access eye care services [5]. This seems to suggest that promotive and preventive eye care activities aimed at discouraging TEM use must have them as prime targets. The high incidence of eye diseases observed in the productive age group has adverse economic implications for the patient, the family, and the country. The economic costs of the resulting visual loss, in this productive age group, arise from direct job losses suffered by the patients, cost of treatment, rehabilitation, and employing care giver for the irreversibly blind [17,18]. The married participants and those residing in rural areas were observed to have a significantly higher likelihood to use TEM than their single and urban counterparts. This is consistent with the findings of previous studies done in Nigeria [2,8]. The influence of marriage probably reflects the role of family members as major prescribers of TEM after traditional medical practitioners [11,19,20]. Rural residence imposes both geographic and economic barriers to access eye care services, which at present, in Nigeria, are concentrated in urban areas; this leaves the rural dwellers with no other alternative eye care provider except the traditional medical practitioners who reside with them in the rural areas [2,8,21]. The higher tendency to use TEM among rural dwellers, which this study has established, implies either rural non-availability or reduced uptake of available promotive and preventive eye care services in the rural areas. This survey did not show any significant association between educational status and TEM use. This is at variance with the findings of in Malawi [11]; however, the difference in the study populations between the two surveys might explain the apparent discrepancy. By implication, health education and health awareness creation programmes, rather than provision of traditional western education, impact more on TEM use and are therefore imperative for creating a positive change in this direction.

The profile of TEM use in this study showed that more of plant products and chemical substances than animal products were used as TEMs. This is consistent with the reports in India [1] and elsewhere [22], and in keeping with the general trend that, in Africa, TEMs are more of plant than animal origin [1]. In addition to traditional African trend, the study area, South-eastern Nigeria, has of late witnessed massive proliferation of spiritual churches (prayer houses), where spiritual healers prescribe partially processed plant products, holy water, and olive oil as pharmacologic adjuncts to their spiritual therapy for eye diseases. The majority (59.7%) of the TEM users obtained their prescriptions from non-traditional practitioners who were patients themselves, friends or relations. This trend in prescription pattern of TEM is similar to observation in Malawi [11] (non-traditional medical practitioner -72.6%, traditional medical practitioner-27.4%)[11]. This implies that, although the traditional medical practitioner is the originator of TEM therapy, societal input plays a crucial role in the perpetuation of the practice. Reported therapeutic benefit from other TEM users and patients' belief in the potency of TEM contributed more to the decision to use TEM than cost and awareness barriers to access orthodox eye care. Contrarily, preference and proximity [11], and absence of side effects and low cost [8], have been previously reported as overriding reasons for using TEM. The scenario spotlights a potential point for intervention in future planning of the eye health literacy needs of the society necessary to curtail the use of TEM. Although the present survey did not document any adverse effect of TEM, their use may constitute a great hazard to the eye even though there are probably some definite therapeutic benefits inherent in their use [23-27]. This suggests that products used as TEM should be subjected to analytical research to isolate, purify, and characterize their active contents for possible use in allopathic medicine. At presentation, 65.8% of TEM users had been on the treatment for eight weeks or less; the treatment modality was mainly direct instillation into the conjunctival sac (84.6%), and 80.5% had abandoned the treatment due to lack of improvement or worsening of eye condition. However, these figures have to be interpreted against the background that 40.0% of TEM users either do not disclose it at all or withhold relevant information relating to its use [10]. That recognized, the duration of therapy, and its abandonment or otherwise, reported by TEM users, in this study, might not have reflected the true situation.

Furthermore, although not reported by any participant, adverse reactions resulting primarily from TEM or from its interaction with prescribed medicines could have contributed to the high abandonment rate. This is corroborated by reported adverse effects of complementary and alternative medicine and their interaction with prescribed medicines [3,5,10].

The clinical profile of the study population revealed that the pattern of presenting complaint did not differ between TEM users and non-users, however, those who reported TEM use presented later, had more of ocular anterior segment pathology, and lower entry distant visual acuity. Late presentation and low presenting visual acuity among TEM users, apparently due to delays caused by prior TEM use, have been variously reported [3,7,11-14]. The spectrum of presenting complaints necessitating presentation in the present survey differed from the series reported in Malawi [11], who had trauma/posterior segment disease topmost, probably due to differences, between the two surveys in survey population and study settings. This suggests that the necessary human and material resources, needed for the treatment of these leading eye conditions, should be made available and accessible to all by planners and implementers of eye care programmes. In this survey, TEM use was significantly associated with co-morbid chronic systemic medical disease. The use of complementary and alternative medicines, similar to TEM, for chronic medical diseases has been reported in children [28] and adults [29]. Perhaps, this may explain the high rate of TEM use among our patients with co-morbid chronic systemic medical diseases. The prevalence of TEM use is a better way of knowing the total population of ophthalmic patients using TEM, unfortunately, we have evaluated only the incidence of TEM use since our focus was on newly presenting ophthalmic patients only. Thus our study was biased towards underestimating TEM use.

The tendency for TEM users to conceal the fact of TEM use or information relating to it, probably to avoid social stigmatisation, could have lead to further underestimation of incidence of TEM use, or affected the reported roles of its correlates.

## Conclusion

The incidence of TEM use among new ophthalmic outpatients at the University of Nigeria Teaching Hospital, Enugu, is low. Chemical substances and plant products rather than animal products were more frequently used as TEM. Non-traditional medicine practitioners were the main prescribers of these TEMs. To reverse the trend, the authors suggest strengthening of promotive and preventive eye care programmes, even distribution of eye care resources, active and continuous collaboration with traditional medical practitioners, and intensification of pharmacological research efforts, to establish the efficacy or otherwise, of the of the "supposedly potent" TEMs.

## Competing interests

The authors declare that they have no competing interests.

## Authors' contributions

BIE:**-**conception and design of study, acquisition, analysis and interpretation of data, drafting of manuscript. CMCO & JNU: - acquisition, analysis and interpretation of data, revising the manuscript for substantial intellectual content and final approval of manuscript for submission. All authors read and approved the final manuscript.

## About the Authors'

Department of Ophthalmology, University of Nigeria Teaching hospital, Ituku-Ozalla, Enugu.

## Pre-publication history

The pre-publication history for this paper can be accessed here:



## Supplementary Material

Additional file 1 **Survey questionnaire/proforma**. The survey questionnaire and proforma used for collecting participants' socio-demographic, historical, and clinical data respectively. Click here for file
