# Supplementary material for: Traditional eye medicine use by newly presenting ophthalmic patients to a teaching hospital in south-eastern Nigeria: socio-demographic and clinical correlates
Source: BMC Complement Altern Med. 2009 Oct 24;9:40. doi: 10.1186/1472-6882-9-40 (PMC2773756; doi:10.1186/1472-6882-9-40)
Supplement: Additional file 1 — Survey questionnaire/proforma. The survey questionnaire and proforma used for collecting participants' socio-demographic, historical, and clinical data respectively. [file 1472-6882-9-40-S1.DOCX]

DEPARTMENT OF OPHTHALMOLOGY, UNIVERSITY OF NIGERIA TEACHING HOSPITAL, ITUKU-OZALLA, ENUGU

**DEPARTMENTAL RESEARCH**

**Survey Questionnaire**

**TOPIC**: Traditional Eye Medicine Use by Newly Presenting Ophthalmic Patients to a              Teaching Hospital in South-eastern Nigeria: Socio-demographic and Clinical              Correlates.

**PARTICIPANTS:** All new eye patients presenting at the ophthalmic outpatient clinic.

**INSTRUCTIONS:** 1.Tick good (√) against the correct response/s in the spaces provided.

2. Provide additional information as appropriate in the spaces provided

**SECTION A: SOCIO-DEMOGRAPHIC CHARACTERISTICS**

1. Sex: (a) Male ( )          (b) Female ( )
2. Age ( years/months/weeks) ______________________
3. Marital status: (a) Single ( )                            (b) Married ( )                             (c) Divorced ( )                             (d) Separated ( )                             (e) Widowed ( )                              (h) Minor(under age) ( )
4. Occupation: (a) Civil servant ( )

(b) Trading ( )

(c) Farming ( )

(d) Artisan ( )

(e)Unemployed ( )

(f) Minor ( )

(f) Retiree ( )

(g) Others specify _________________________________

1. Educational status: (a) Primary ( )                                     (b) Secondary ( )

(c) Tertiary ( )

(d) None ( )

(e) Minor (pre-school age) ( )

6. Residence: (a) Urban ( )                                             (b) Rural ( )

**SECTION B: CLINICAL PROFILE**

7. Main presenting ocular complaint: ________________________________

8. Duration of main presenting ocular complaint: ______________________

9. Best corrected distant visual acuity: (a) Right eye __________ (b) Left eye ____________

10. Main definitive clinical diagnosis: (a) Right eye ___________________________                                                          (b) Left eye____________________________

11.Location of ocular pathology : (a) Anterior segment ( )                                                             (b) Posterior segment ( )                                                             (c) Anterior and posterior segments ( )      (d) Extra-ocular ( )

12. Associated medical conditions. (a) HBP ( ) (b) DM ( ) (c) HIV/AIDS ( ) (d) cancer ( ) (e)       Asthma ( ) (f) Others Specify_________________________

13. Are you on prescribed medicine (a) Yes ( ) (b) No ( )

**SECTION C: PROFILE OF TEM USE** 14. Nature of TEM: _________________________________

15. Route of administration of TEM:(a) Topical ( )                                                                  (b) Oral ( )                                         (c) Face wash ( )                                                                  (d) Inhalation ( )                                                                  (e) Per aural ( )                                                                  (f) Injection ( )                                                      (g) Fume bath ( )                                                                  (h) Shower ( )     (I ) Instillation ( )                                                                  (j ) Others specify_____________________________

16. Prescriber of TEM: **Traditional medical practitioner**         (a) Traditional Healer ( )      (b) Clergyman ( )   (c) Alfa ( )                                          **Non-traditional medical practitioner**     (c) Patient ( )    (d) Friend ( )    (e) Relation( )                            (f) Do not know ( )        (g) Others specify __________________________

17. Main reason for TEM use: (a) Others benefited ( )                                             (b) Belief in potency ( )                                               (c) Orthodox medicine un-affordable ( )                                               (d) Unaware of orthodox alternatives ( )                                               (e) Unsatisfactory orthodox treatment ( )                                               (f) Others specify ______________________

18. Duration of TEM use (years/months/weeks): __________________________

19. Cessation of TEM use: (a) Yes ( )                   (b) No ( )

20. If yes in No 15, what is your main reason: (a) No improvement ( )                                                  (b) Condition worsening ( )     (c) Advised to stop ( )                        (d) Intolerant of TEM ( )                                                                               (e) Adverse reaction ( )                   (f) Others specify______________

21. Any adverse interaction with prescribed medicines (a) Yes (b) No ( )

Thank you
